# Prediction of some milk production traits using udder and teat measurements with a spotlight on their genetic background in Friesian cows

**DOI:** 10.1038/s41598-023-43398-y

**Published:** 2023-09-27

**Authors:** Ahmed. A. Saleh, Ahmed A. Easa, Dalia K. EL-Hedainy, Amr M. A. Rashad

**Affiliations:** 1 Animal and Fish Production Department, Faculty of Agriculture (El-Shatby), Alexandria University, Alexandria, 22545 Egypt; 2 Animal and Poultry Production Department, Faculty of Agriculture, Damanhour University, Damanhour, 22511 Egypt

**Keywords:** Animal breeding, Biotechnology, Zoology

## Abstract

The aggregate udder shape (Bowl, Round, Cup), udder measurements (udder width, UW, udder front depth, UFD, udder rear depth, URD, udder levelness, ULV, udder heights, UH) and teat measurements (teat diameters, TD, front teat length, FTL, rear teat length, RTL, distance between front teats, DFT and distance between rear teats, DRT) were measured on 1300 Friesian cows located in a commercial farm under subtropical conditions (Egypt) to appraise udder and teats status and to evaluate the possible relationships with some milk production characteristics in conjunction with udder shape, age at first calving, sire and inbreeding effects on udder morphological traits and milk production ability. For such an available sample size, parity had affected (*P *< 0.01) UFD, TD, FTL and RTL. In addition, udder shape affected (*P *< 0.01) UW, UFD, URD, ULV and DFT. None of the other studied factors affected milk production traits. The bowl udder shape (*P *< 0.01) yielded a high total milk yield (3267.19 kg), adjusted milk yield (2443.01 kg) and lactation length (480.70 d) compared to other udder shapes. The genetic correlations of UW with total milk yield and persistency were strongly positive (0.86 and 0.93, respectively). However, strong negative genetic correlations were found between UW with peak milk yield and lactation length (− 0.92 and − 0.80, respectively), between RTL with peak milk yield (− 0.92) and DRT with persistency (− 0.79). As found from the stepwise multiple regression, UW and URD can be used as good indicators for predicting milk yield and lactation length. Additionally, this study spotlights the genetic background of udder characteristics based on reliable studies and the QTL database for cattle as a first step toward applying this knowledge side by side with phenotypic traits to improve the productivity of the Holstein breed under subtropical conditions.

## Introduction

Concerning the productivity evaluation of dairy cattle, udder conformation are important elements for the assessment of milk production^1,2^. The conformation of cow udder used to be one of the important criteria for predicting milk production performance^3,4^. Udder measurements are important for the prognosis of udder health status and functionality of milk production in dairy cows. The morphology and anatomy of cow udder has long been a subject for curious selection to improve the efficiency of milk production^5^. In addition, the teats of dairy animals are an important part of the udder, which is attached with a milking cluster and meantime, serves the roles of both a valve regulating the outflow of milk and of a natural barrier for exogenous infections^6,7^, teat shape and dimensions have no less emphasis in that regard^5^. Poor udder and teat conformations form a management challenge for dairy cow commercial producers. However, selection against poor teats and udders increases the profit potential by increasing performance, longevity and showing the ability of the cow^8–11^. Moreover, significant relationships were monitored between dimensions and formation of teats and teat canal length with milk-ability and milk flow speed traits^12,13^.

Bhuiyan et al.^14^ reported that udder size and shape conformation traits could play a vital role in the suitability for easy milking and economical milk production and should be considered when selecting dairy cows. A large sized udder with a large proportion of glandular tissue and a symmetrical shape is an asset to a milk animal^15,16^. Seykora and McDanel^17^ found that udder depth and teat-end shape have been associated with udder health and reducing frequencies of cows with deep udders and flat disks or inverted teat ends may reduce mastitis incidence. In addition, streak canal diameter was negatively correlated with udder health, though it is difficult to measure^18^.

Many researchers agree that there is a linear relationship between teat distances and milk yield^19–21^ and that the teat canal length possesses a significant effect on the flow of milk through the teat canal^22^. Klein et al.^23^ recommend long and narrow teats to improve udder health. The teat canal length had a significant effect on milk flow characteristics such as daily milk yield, average milk flow, maximum milk flow and somatic cell score, at different measurement times^24,25^. Additionally, the length and morphology of the teat canal may have an effect on the development of udder infection and defence mechanisms^25^. Factors that have been associated with the quality of these mechanisms are udder depth, fore udder attachment, teat length, teat shape, and milk-ability^26^. It is likely that high udders with good attachments and small teats are less prone to teat lesions^27^. Relationships between teat shape and measurements with milk yield should be regarded as criteria for the selection of dairy cattle^19,28^. Wilmink^29^ and Sabuncuoglu et al.^30^ proposed that in dairy cattle selection, conformation traits, udder depth, suspension of fore quarters and teat location should be considered in addition to milk performance. Correlations between traits describing udder form and milk performance indicate clearly that udder depths, distance to floor, hind udder attachment, udder band, teat placement and teat size are all of economic relevant and useful to describe udder functionality. These traits can be recorded with a high degree of accuracy and repeatability^31,32^. Type traits are recorded relatively early in life of animals and are medium to highly heritable^26,33^. Heritability of udder morphology is to high^34^, a single score during the lifetime of a cow may be adequate for selection and which makes selection relatively easier and more efficient. Udder height was a good predictor of lactation performance^35^. Moreover, differences in udder shape and size were reported to be heritable^15^. Daughters of highly proven milk bulls possessed greater distance between teats, greater perimeters and larger areas of the udder floor and udder length were significantly correlated with milk yield^36^. Most dairy cattle breeding objectives worldwide have focused exclusively on production traits paying no attention to the severe deterioration in production properties caused, mainly by the unfavourable phenotypic and genetic correlations between linear type and milk production traits^37^. On the other side, there are genomic regions, that involve quantitative traits locus (QTLs), candidate genes and significant single-nucleotide-polymorphisms (SNPs) related to the udder and teat traits in dairy cattle^6,26,38^.

The present study aimed to evaluate udder and teat characteristics in order to predict their relationship with milk production traits. The effects of udder shape, sire, parity, inbreeding and age at first calving on udder and teats measurements were also studied, with a spotlight on the genetic background of concerning traits.

## Materials and methods

This experimental protocol was approved by the Ethics Committee of Damanhur University under approval number DFU-2023-2.

### Description of data

This study was conducted on 1300 pure Friesian cows located in a commercial farm (GPS; 30.30439731069514, 30.44699928913166), in Wadi-El-Natrun, Al-Beheira Governorate, Egypt. Cows were housed free in open semi-shaded yards, nourished under the prevailing feeding conditions according to the NRC requirements and milked twice daily.

### Traits of concern

**Figure 1:**
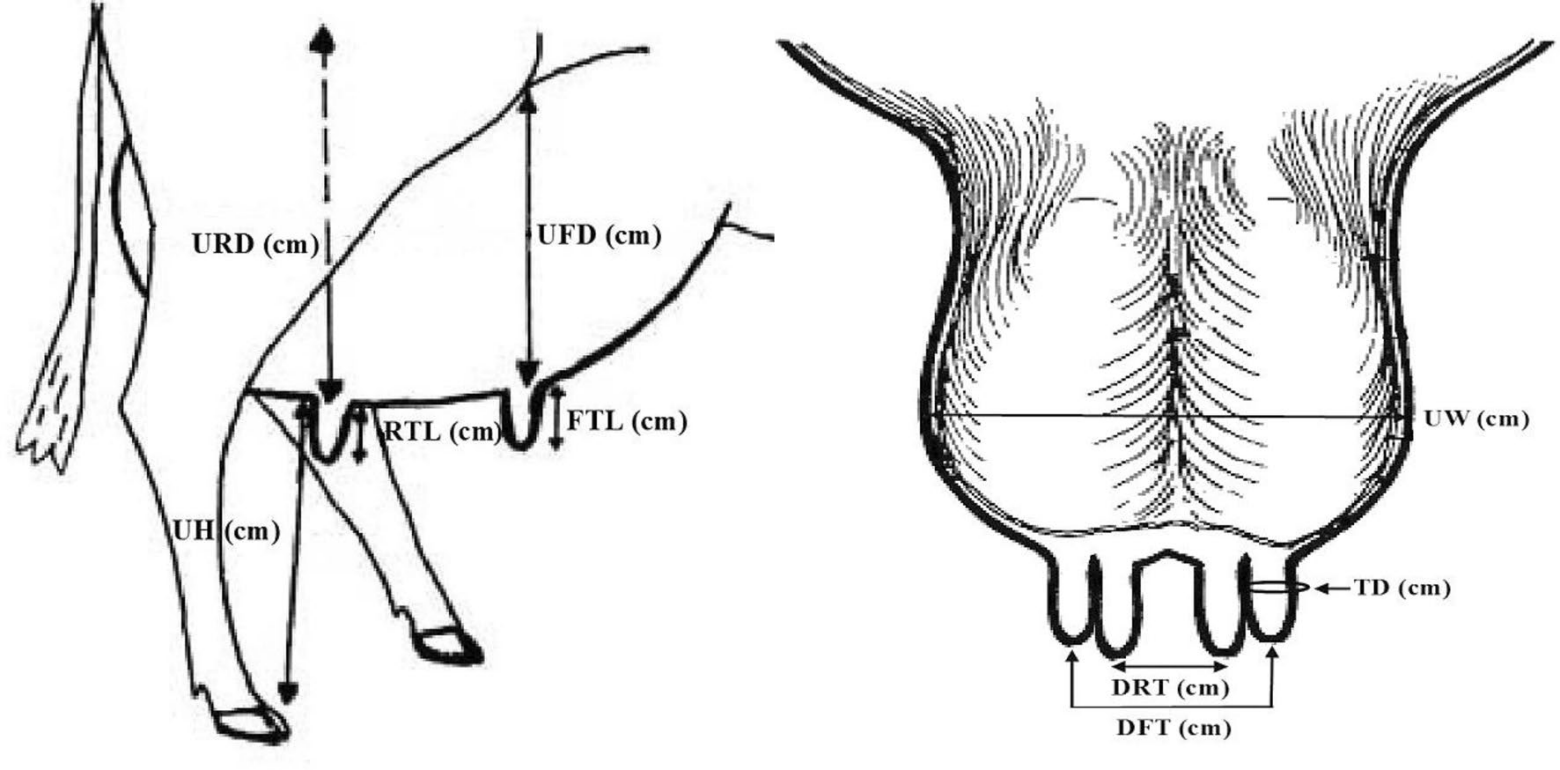
Udder and teat characteristics of Friesian cows (UW: udder width, UFD: udder front depth, URD: udder rear depth, UH: udder heights, TD: teat diameters, FTL: front teat length, RTL: rear teat length, DFT: distance between front teats and DRT: distance between rear teats).

The udder and teat measurements (in cm) were taken monthly on a fixed day throughout the lactation period one hour before the evening milking. The udder and teat measurements were udder width (UW); distance between right and left sides of the udder at the widest point, udder front depth (UFD); distance from the merging point of fore udder with the abdomen to a point in front of the fore teats at the level of the udder base, udder rear depth (URD); distance from the bottom of the vulva to the base of the rear udder, udder levelness (ULV); the difference between the rear and front udder heights^39^, udder heights (UH); distance from the ground to udder floor in front of the front or behind rear teat, teat diameters (TD); diameters of front teat or rear teat measured at mid-point of each teat length from teat orifice to base of udder, front teat length (FTL); length of front teat from teat orifice to base of udder, rear teat length (RTL); length of rear teat from teat orifice to base of udder, distance between front teats (DFT); distances between front teats before milking and at mid teat length, distance between rear teats (DRT); distances between rear teats before milking and at mid teat length (Fig. 1).

Recorded milk production traits were total milk yield (TMY, kg), adjusted milk yield (305d-MY, kg), peak milk yield (PMY, kg), lactation length (LL, day) and persistency (PR, %) which was calculated according to Lean et al.^40^ as ratio of 305d-MY / (PMY * 305). Udders were classified into bowl, cup and round shapes.

### Genetic background of udder and teat measurements

According to reliable investigations, many genome regions, candidate genes, SNPs and QTLs have been reported to be correlated with several udder characteristics in different species including cattle. In this regard, the genetic background for udder characteristics in the cattle has been investigated utilizing; (1) FAO-Database (https://www.fao.org), (2) Animal QTL-Database: (https://www.animalgenome.org/cgi-bin/QTLdb/BT/index) and (3) Genome-Informatics-Resources: (https://www.animalgenom e.org/bioinfo) for further and complementary future studies.

### Statistical analysis

All sets of data were tested for normality with the Shapiro–Wilk test from the UNIVARIATE procedure SAS 9.0 (2009)^41^, and results indicated that all data were distributed normally (W ≥ 0.90). To study the factors affecting udder or teat characteristics, MIXED procedure of SAS 9.0 (2009)^41^ was used according to the following model:

$$ {\mathbf{Y}}_{{{\mathbf{ijklmn}}}} ={\varvec{\upmu}} + {\mathbf{S}}_{{\mathbf{i}}} + {\mathbf{P}}_{{\mathbf{j}}} + {\mathbf{I}}_{{\mathbf{k}}} + {\mathbf{U}}_{{\mathbf{l}}} + {\mathbf{b}}_{{\mathbf{m}}} \left( {{\mathbf{x}} - \overline{x}} \right) + {\mathbf{ e}}_{{{\mathbf{ijklmn}}}} $$

where: **Y**_**ijklmn**_ = any of the udder or teat measurements, **µ** = the overall mean, **S**_**i**_ = the random effect of ith sire (i = 1:180), **P**_**j**_ = the fixed effect of jth parity (j = 1:5), **I**_**k**_ = the fixed effect of kth inbreeding (inbred or non-inbred), **U**_**l**_ = the fixed effect of lth udder shape (bowel, cup and round), **b**_**m**_ = a regression coefficient of each udder and teat measurement on the independent continuous variable of age at first calving (AFC, x)specific to the teat or udder measurement and $\overline{x}$ = the respective mean, and **e**_**ijklmn**_ = the residual error.

Significant differences among means within each factor were tested using Duncan test. The heritability values of each udder or teat measurement was estimated using variance components (Proc VARCOMP, SAS 2009)^41^ according to the following equation^42^:

$$ {\mathbf{h}}^{{\mathbf{2}}} = {\mathbf{4}}{\varvec{\upsigma}}^{{\mathbf{2}}} {\mathbf{S}}/\left( {{\varvec{\upsigma}}^{{\mathbf{2}}} {\mathbf{S}} + {\varvec{\upsigma}}^{{\mathbf{2}}} {\mathbf{e}}} \right) $$

where; **σ**^**2**^**S** = the variance of sire and **σ**^**2**^**e** = the variance of error. Simple regressions and correlations between udder or teat measurements and all milk production traits were calculated. Besides, the stepwise multiple regression analysis of milk production traits on udder and teat measurements were calculated using STEPWISE procedure of SAS^41^ to determine the best regression model.

### Ethical approval

The study was performed in accordance with the ethical standards as laid down in the 1964 Declaration of Helsinki and its later amendments or comparable ethical standards. Also, this experimental protocol was approved by the Ethics Committee of Damanhur University under approval number DFU-2023-2.

## Results

### Effect of sire, inbreeding, parity, udder shape and age at first calving on udder and teat measurements

Means, standard deviations and coefficients of variation of udder and teat characteristics in different udder shape groups are shown in Table 1. All characteristics were varied from shape to other, bowl shape had highest udder measurements (UW, UFD, URD and ULV) expect (UH). On the contrary cup shape had lowest udder measurements (UW, UFD, URD and ULV) expect (UH) which were high. The variation in udder measurements (UW, UFD and URD) between udder shapes ranged from 9.62 to 21.27% expect ULV (cm) which had the highest variation (C.V. = − 76.70). Bowl shaped udder had highest teat diameters and distance between front teats (2.79 and 15.25 cm, respectively) while the round shape had highest front teat length and rear teat length (7.03 and 6.78 cm, respectively). On the other hand, the cup shaped udder had highest distance between rear teats (8.63 cm).

**Table 1:** Means, standard deviation (SD) and coefficient of variation (CV) for udder and teat measurements in different udder shapes of Friesian cows.

Measurement^a^ (cm.)	Udder shape							Overall (no = 1300)		
	Bowl (no = 320)			Round (no = 580)		Cup (no = 400)				
	Mean		SD	Mean	SD	Mean	SD	Mean	SD	CV %
Udder										
UW	39.84		1.80	32.98	2.67	23.28	2.75	31.68	6.74	21.27
UFD	23.19		2.67	19.02	2.80	15.60	2.56	18.99	3.88	20.46
URD	28.38		3.37	22.67	2.74	18.48	3.49	22.78	4.82	21.15
ULV	− 5.19		3.91	− 3.66	2.27	− 2.88	2.42	− 3.79	2.91	− 76.78
UH	55.09		7.01	57.52	4.20	57.58	5.53	56.94	5.48	9.62
Teat										
TD	2.79		0.47	2.75	0.47	2.68	0.37	2.74	0.44	16.08
FTL	6.50		1.38	7.03	1.24	6.98	1.08	6.88	1.24	18.05
RTL	6.50		1.45	6.78	1.25	6.76	1.22	6.70	1.29	19.25
DFT	15.25		1.97	14.59	1.83	14.83	2.56	14.82	2.11	14.24
DRT	8.13		2.35	8.48	2.62	8.63	2.32	8.44	2.45	29.07

^a^*UW* Udder width, *UFD* Udder front depth, *URD* Udder rear depth, *ULV* Udder levelness, *UH* Udder height, *TD* Teat diameter, *FTL* Front teat length, *RTL* Rear teat length, *DFT* Distance between front teats, *DRT* Distance between rear teats.

Least squares means and standard errors for UW, UFD, URD, ULV and UH as affected by sire, inbreeding status, parity, udder shape and age at first calving are presented in Table 2. Sire, inbreeding status and age at first calving had no effects (*P* > 0.01) on UW, UFD, URD, ULV or UH, but parity had an effect (*P* < 0.01) on UFD which was the highest (22.58 cm) in parity 3. Udder shape had effects (*P* < 0.01) on UW, UFD, URD and ULV with highest values of UW, UFD and URD (38.47, 23.65 and 28.91, respectively) obtained for bowl shaped udders but the highest values of ULV was obtained in cases of round (− 3.48 cm) and cup shaped (− 3.03 cm).

**Table 2:** Effect of sire, inbreeding, parity, udder shape and age at first calving on udder measurements (cm) for Friesian cows (LSM ± SE).

Factor	No.	UW	UFD	URD	ULV	UH
Sire	(180)^1^	ns	ns	ns	ns	ns
Min		30.30 ± 1.30	15.25 ± 1.58	20.54 ± 1.19	− 6.11 ± 1.14	50.67 ± 3.60
Max		34.70 ± 1.70	22.28 ± 1.31	26.09 ± 1.58	− 0.34 ± 1.11	60.13 ± 2.22
Inbreeding		ns	ns	ns	ns	ns
Non inbred	650	33.04 ± 0.34	20.27 ± 0.53	23.89 ± 0.61	− 3.62 ± 0.57	54.66 ± 1.06
Inbred	650	32.38 ± 0.38	20.13 ± 0.52	24.35 ± 0.60	− 4.22 ± 0.56	55.88 ± 1.05
Parity		ns	**	ns	ns	ns
1	580	31.41 ± 0.41	17.51^c^ ± 0.60	22.19 ± 0.69	− 4.68 ± 0.64	57.23 ± 1.18
2	370	32.87 ± 0.87	18.97^b^ ± 0.56	24.34 ± 0.64	− 5.36 ± 0.60	56.87 ± 1.07
3	130	32.39 ± 1.39	22.58^a^ ± 1.01	24.97 ± 1.16	− 2.39 ± 1.08	54.07 ± 1.96
4	120	33.18 ± 1.18	20.60^ab^ ± 1.11	25.50 ± 1.28	− 4.90 ± 1.20	54.40 ± 2.21
5 and over	100	33.70 ± 1.70	21.33^ab^ ± 1.17	23.61 ± 1.35	− 2.28 ± 1.26	53.79 ± 2.33
Shape		**	**	**	**	ns
Bowl	320	38.47^a^ ± 0.90	23.65^a^ ± 0.57	28.91^a^ ± 0.66	− 5.26^b^ ± 0.62	56.04 ± 1.91
Round	580	33.46^b^ ± 0.64	19.92^b^ ± 0.53	23.40^b^ ± 0.61	− 3.48^a^ ± 0.57	56.01 ± 1.36
Cup	400	26.21^c^ ± 0.92	17.02^c^ ± 0.63	20.05^c^ ± 0.72	− 3.03^a^ ± 0.68	53.76 ± 1.95
Reg. on age at first calving^2^	1300	ns	ns	ns	ns	ns
		(0.15 ± 0.14)	(0.11 ± 0.08)	(0.14 ± 0.11)	(− 0.02 ± 0.06)	(0.18 ± 0.11)

*UW* Udder width, *UFD* Udder front depth, *URD* Udder rear depth, *ULV* udder levelness, *UH* Udder height, *ns* Not significant.

^1^Number of sire.

^2^Number between practices: simple regression (b ± SE).

^a^^–c^Least Squares Means with different letters in the same column are significantly different.

**Significant at *P* < 0.01.

Least squares means and standard errors for TD, FTL, RTL, DFT and DRT of sire, inbreeding status, parity, udder shape and age at first calving are presented in Table 3. Sire, inbreeding status and age at first calving had no effect (*P* > 0.01) on TD, FTL, RTL, DFT and DRT. Parity had effects (*P* < 0.01) on TD, FTL and RTL with the highest values obtained in parities 3, 4 and 5. Udder shape had no effects on teat measurements except DFT which was the highest (*P* < 0.01) for cup shaped udder (16.41 cm).

**Table 3:** Effect of sire, inbreeding, parity, udder shape and age at first calving on teat measurements (cm) for Friesian cows (LSM ± SE).

Factor	No.	TD	FTL	RTL	DFT	DRT
Sire	(180)^1^	ns	ns	ns	ns	ns
Min		2.00 ± 0.42	6.25 ± 0.50	5.84 ± 0.53	13.61 ± 2.01	6.60 ± 1.04
Max		3.62 ± 0.26	8.81 ± 0.70	8.50 ± 0.75	17.63 ± 1.24	12.15 ± 1.21
Inbreeding		ns	ns	ns	ns	ns
Non inbred	650	3.07 ± 0.09	7.49 ± 0.26	7.28 ± 0.27	15.49 ± 0.43	8.72 ± 0.48
Inbred	650	2.99 ± 0.09	7.76 ± 0.26	7.37 ± 0.27	15.33 ± 0.42	8.71 ± 0.48
Parity		**	*	**	ns	ns
1	580	2.39^b^ ± 0.10	6.32^b^ ± 0.29	5.96^b^ ± 0.30	14.41 ± 0.47	8.63 ± 0.54
2	370	2.67^b^ ± 0.09	6.87^b^ ± 0.26	6.56^b^ ± 0.27	14.79 ± 0.43	8.73 ± 0.48
3	130	3.22^a^ ± 0.17	8.01^a^ ± 0.48	7.78^a^ ± 0.50	15.17 ± 0.78	8.18 ± 0.89
4	120	3.37^a^ ± 0.19	8.50^a^ ± 0.54	8.32^a^ ± 0.56	16.79 ± 0.88	7.60 ± 1.00
5 and over	100	3.51^a^ ± 0.20	8.42^a^ ± 0.57	7.99^a^ ± 0.60	15.88 ± 0.93	10.41 ± 1.06
Shape		ns	ns	ns	*	ns
Bowl	320	2.80 ± 0.16	6.82 ± 0.47	6.65 ± 0.49	15.41^ab^ ± 0.76	6.81 ± 0.87
Round	580	3.11 ± 0.12	7.84 ± 0.33	7.60 ± 0.35	14.41^b^ ± 0.54	9.17 ± 0.62
Cup	400	3.19 ± 0.17	8.22 ± 0.48	7.71 ± 0.50	16.41^a^ ± 0.78	10.16 ± 0.89
Reg. on age at first calving^2^	1300	ns	ns	ns	ns	ns
		(− 0.02 ± 0.01)	(− 0.09 ± 0.02)	(− 0.03 ± 0.02)	(− 0.06 ± 0.04)	(− 0.06 ± 0.05)

*TD* Teat diameter, *FTL* Front teat length, *RTL* Rear teat length, *DFT* Distance between front teats, *DRT* Distance between rear teats, *ns* Not significant.

^1^Number of sire.

^2^Number between practices: simple regression (b ± SE).

^a^^–c^Least Squares Means with different letters in the same column are significantly different.

*Significant at *P* < 0.05.

**Significant at *P* < 0.01.

### Effect of sire, inbreeding, parity, udder shape and age at first calving on milk production traits

From Table 4, all milk production traits were not affected (*P* > 0.05) by any of the studied factors except udder shape which affected (*P* < 0.01) TMY, 305d-MY and LL but not did for PMY and PR. Bowl udder produce the highest (*P* < 0.01) TMY and 305d-MY which were 3267.19 and 2443.01 kg and had the longest (*P* < 0.01) LL being 480.70 day.

**Table 4:** Effect of sire, inbreeding, parity, udder shape and age at first calving on milk production traits for Friesian cows (LSM ± SE).

Factor	No	TMY, kg	305d-MY, kg	PMY, kg/day	LL, day	PR, %
Sire	(180)^1^	ns	ns	ns	ns	ns
Min		1853.87 ± 305.65	1831.05 ± 215.11	8.85 ± 1.39	241.15 ± 56.29	50.35 ± 9.64
Max		2903.31 ± 254.74	2324.60 ± 355.96	11.68 ± 1.12	415.23 ± 47.40	76.35 ± 8.17
Inbreeding		ns	ns	ns	ns	ns
Non inbred	650	2297.25 ± 101.17	2121.53 ± 72.37	10.91 ± 0.46	307.15 ± 18.83	60.44 ± 3.24
Inbred	650	2328.15 ± 99.40	2125.86 ± 71.10	10.20 ± 0.45	313.58 ± 18.50	63.64 ± 3.19
Parity		ns	ns	ns	ns	ns
1	580	2333.77 ± 114.38	2085.98 ± 81.82	8.86 ± 0.52	326.22 ± 21.29	69.22 ± 3.67
2	370	2328.49 ± 104.01	2151.63 ± 74.40	9.21 ± 0.47	293.76 ± 19.35	69.24 ± 3.34
3	130	2339.56 ± 186.60	2113.83 ± 133.47	11.46 ± 0.85	333.43 ± 34.72	62.45 ± 5.98
4	120	2240.51 ± 214.51	2119.81 ± 153.44	10.99 ± 0.97	289.46 ± 39.92	58.56 ± 6.88
5 and over	100	2321.16 ± 217.87	2147.20 ± 155.85	12.26 ± 0.99	308.96 ± 40.54	50.72 ± 6.99
Shape		**	**	ns	**	ns
Bowl	320	3267.19 ± 183.75^a^	2443.01 ± 131.44^a^	11.39 ± 0.83	480.70 ± 34.19^a^	59.23 ± 5.89
Round	580	2266.59 ± 122.72^b^	2257.44 ± 87.78^b^	10.31 ± 0.56	297.27 ± 22.84^b^	67.84 ± 3.94
Cup	400	1404.31 ± 190.77^c^	1670.62 ± 136.46^c^	9.96 ± 0.87	153.13 ± 35.50^c^	59.04 ± 6.12
Reg. on age at first calving^2^	1300	ns	ns	ns	ns	ns
		(10.98 ± 21.63)	(11.71 ± 15.96)	(0.71 ± 0.54)	(3.13 ± 0.59)	(1.14 ± 0.88)

*TMY* Total milk yield, *305d-MY* Adjusted milk yield for 305 day, *PMY* Peak milk yield, *LL* Lactation length, *PR* Persistency, *ns* Not significant.

^1^Number of sire.

^2^Number between practices: simple regression (b ± SE).

^a^^–c^Least Squares Means with different letters in the same column are significantly different.

**Significant at *P* < 0.01.

### Relationships between udder or teat measurements and some milk production traits

Estimates of heritability and genetic correlation for udder and teat measurements with milk production traits are summarized in Table 5. The estimated Heritability for the reproduction traits were close to zero. Heritability estimates ranged between 0.464 (for TD) and 0.103 (for UH). The genetic correlations between TMY, 305d-MY, Peak, LL and PR with udder and teat measurements are presented in Table 5. UW had strong positive genetic correlation with 305d-MY and PR (0.86 and 0.93). Also, UH has similar strong positive correlation with PR (0.87). On the other hand, strong negative genetic correlations were found between UW with both PMY and LL (− 0.92 and − 0.80, respectively) and between UFD with PR (− 0.72), URD with PMY (− 0.84), RTL with PMY (− 0.92) and DRT with PR (− 0.79). This reflects a negative relationship between these traits.

**Table 5:** Genetic parameters estimates for udder and teat measurements with some milk production traits.

Traits^a^	Genetic correlation (r_g_)					Heritability (h^2^)
	TMY, kg	305d-MY, kg	PMY, kg	LL, day	PR., %	
Udder (cm)						
UW	− 0.28	0.86	− 0.92	− 0.80	0.93	0.359
UFD	− 0.16	− 0.23	− 0.25	0.21	− 0.72	0.339
URD	− 0.17	− 0.23	− 0.84	0.20	− 0.43	0.254
ULV	− 0.14	− 0.19	− 0.16	0.16	− 0.47	0.401
UH	0.42	− 0.23	− 0.43	0.14	0.87	0.103
Teat (cm)						
TD	0.64	0.66	− 0.18	0.40	− 0.18	0.464
FTL	0.12	0.06	− 0.56	0.15	− 0.14	0.415
RTL	0.12	− 0.29	− 0.92	0.17	− 0.22	0.125
DFT	− 0.37	0.45	− 0.24	− 0.14	0.03	0.394
DRT	− 0.51	0.11	− 0.27	− 0.12	− 0.79	0.416

^a^*UW* Udder width, *UFD* Udder front depth, *URD* Udder rear depth, *ULV* Udder levelness, *UH* Udder height, *TD* Teat diameter, *FTL* Front teat length, *RTL* Rear teat length, *DFT* Distance between front teats, *DRT* Distance between rear teats, *TMY* Total milk yield, *305d-MY* Adjusted milk yield, *PMY* Peak milk yield, *LL* Lactation length, *PR* Persistency.

Simple correlations and regressions of some milk production traits on the examined udder or teat characteristics (cm) are presented in Table 6. UW had the highest (*P *< 0.05) positive correlations with milk production traits and ranged from 0.23 to 0.87. Also, UFD and URD had high (*P *< 0.05) positive correlations with milk production traits except that between URD and PR which were moderate positive but insignificant. The correlations between ULV, UH with milk traits were negative except those with PR which were nearly equal zero (0.03). On the other hand, the correlations between teat measurements and PR were moderate negative (*P *< 0.05). Also, the correlations between teat measurements with TMY, 305d-MY and LL approached zero (*P *> 0.05). Beside, TD, DFT and DRT teats were moderated correlated (*P *< 0.05) with PMY. The same trend was found for regression; udder width had the highest R^2^ values for all milk traits except persistency and ranged from 31.61 to 75.22 followed by URD and UFD. The regression coefficients indicated that, for each 1 cm increase in UW an increase of 110.8 kg (R^2^ = 70.41) in TMY and of 10.79 day (R^2^ = 40.26) increase in LL occurred. Low R^2^ values were found for ULV and UH (ranged from 0.09 to 11.95) and all teat measurements (ranged from 0.06 to 6.95) with milk production traits.

**Table 6:** Simple correlations and regressions of some milk production traits on the examined udder and teat measurements (cm).

Traits	TMY, kg			305d-MY, kg			PMY, kg			LL, day			PR, %		
	r	b	R^2^	r	b	R^2^	r	b	R^2^	r	b	R^2^	r	b	R^2^
Udder (cm)															
UW	0.84*	110.80*	70.41	0.87*	87.2*	75.22	0.56*	0.22*	31.61	0.64*	10.79*	40.26	0.23*	0.49*	4.83
UFD	0.67*	153.92*	45.13	0.71*	123.92*	50.50	0.47*	0.31*	22.24	0.48*	14.21*	23.19	0.23*	0.87*	5.10
URD	0.75*	138.56*	55.95	0.74*	104.51*	55.26	0.47*	0.25*	21.80	0.57*	13.45*	32.00	0.16	0.50	2.67
ULV	− 0.35*	− 105.80*	11.95	− 0.28*	− 65.82*	7.98	− 0.14	− 0.12	2.06	− 0.29*	− 11.59*	8.60	0.03	0.16	0.09
UH	− 0.14	− 23.00	2.00	− 0.12	− 14.30	1.34	− 0.07	− 0.03	0.42	− 0.10	− 2.09	1.00	0.03	0.08	0.10
Teat (cm)															
TD	0.10	204.20	1.02	0.15	235.80	2.35	0.23*	1.32*	5.06	0.03	8.83	0.11	− 0.22*	− 7.32*	4.65
FTL	− 0.08	− 54.70	0.58	− 0.04	− 23.70	0.19	0.09	0.19	0.83	− 0.07	− 6.23	0.45	− 0.17*	− 2.10*	3.03
RTL	− 0.06	− 42.90	0.39	− 0.03	− 15.10	0.08	0.02	0.05	0.06	− 0.07	− 6.03	0.46	− 0.22*	− 2.58*	4.96
DFT	0.12	48.40	1.32	0.14	45.80	2.04	0.26*	0.32*	6.95	0.06	2.96	0.30	− 0.20*	− 1.43*	4.10
DRT	− 0.04	− 13.50	0.14	− 0.03	− 7.20	0.07	0.17*	0.18*	3.03	− 0.08	− 3.64	0.61	− 0.26**	− 1.57*	6.67

*UW* Udder width, *UFD* Udder front depth, *URD* Udder rear depth, *ULV* Udder levelness, *UH* Udder height, *TD* Teat diameter, *FTL* Front teat length, *RTL* Rear teat length, *DFT* Distance between front teats, *DRT* Distance between rear teats, *TMY* Total milk yield, *305d-MY* Adjusted milk yield, *PMY* Peak milk yield, *LL* Lactation length, *PR* Persistency, *a* Intercept, *b* Regression coefficient, *R*^2^ Coefficient of determination.

*Significant at *P* < 0.05.

Stepwise multiple regression analysis was utilized to predict milk traits from udder and teats measurements as shown in Table 7. Indeed, UW still the best predictor for most milk production traits followed by udder rear depth for predicting TMY, 305d-MY and LL (R^2^ = 74.02, 77.43 and 42.26, respectively) and then no other variables met the level of significant. In contrast, teat measurements were entered in equation for predicting PMY and PR but with low change in R^2^. DRT was the first measurement entered for predicting persistency (R^2^ = 6.67) followed by UW with change in R^2^ = 4.97.

**Table 7:** The best stepwise multiple regression equations of milk production traits on udder and teat measurements.

Dependent variable	Intercept (a)	Regression coefficient (b)							R^2^
		UW	URD	TD	FTL	RTL	DFT	DRT	
TMY, kg	− 1248.84	110.83	–	–	–	–	–	–	70.41
	− 1561.15	83.02	52.38	–	–	–	–	–	74.02
305d-MY, kg	− 716.13	87.20	–	–	–	–	–	–	75.22
	− 901.98	70.65	31.17	–	–	–	–	–	77.43
PMY, kg	2.62	0.22	–	–	–	–	–	–	31.51
	− 0.36	0.21	–	–	–	–	0.22	–	34.86
	− 2.45	0.21	–	–	0.29	–	0.22	–	36.76
	− 2.26	0.21	–	–	0.61	− 0.40	0.24	–	38.43
LL, day	− 21.03	10.80	–	–	–	–	–	–	40.26
	− 50.92	8.14	5.01	–	–	–	–	–	42.26
PR, %	79.74	–	–	–	–	–	–	− 1.57	6.67
	64.22	− 1.59	–	–	–	–	–	0.49	11.64
	75.37	0.56	–	− 6.20	−	−	−	− 1.15	14.36

*UW* Udder width, *URD* Udder rear depth, *TD* Teat diameter, *FTL* Front teat length, *RTL* Rear teat length, *DFT* Distance between front teats, *DRT* Distance between rear teats, *TMY* Total milk yield, *305d-MY* Adjusted milk yield, *PMY* Peak milk yield, *LL* Lactation length, *PR* Persistency, *a* Intercept, *b* Regression coefficient, *R*^2^ Coefficient of determination.

### Genetic background of udder and teat measurements

**Figure 2:**
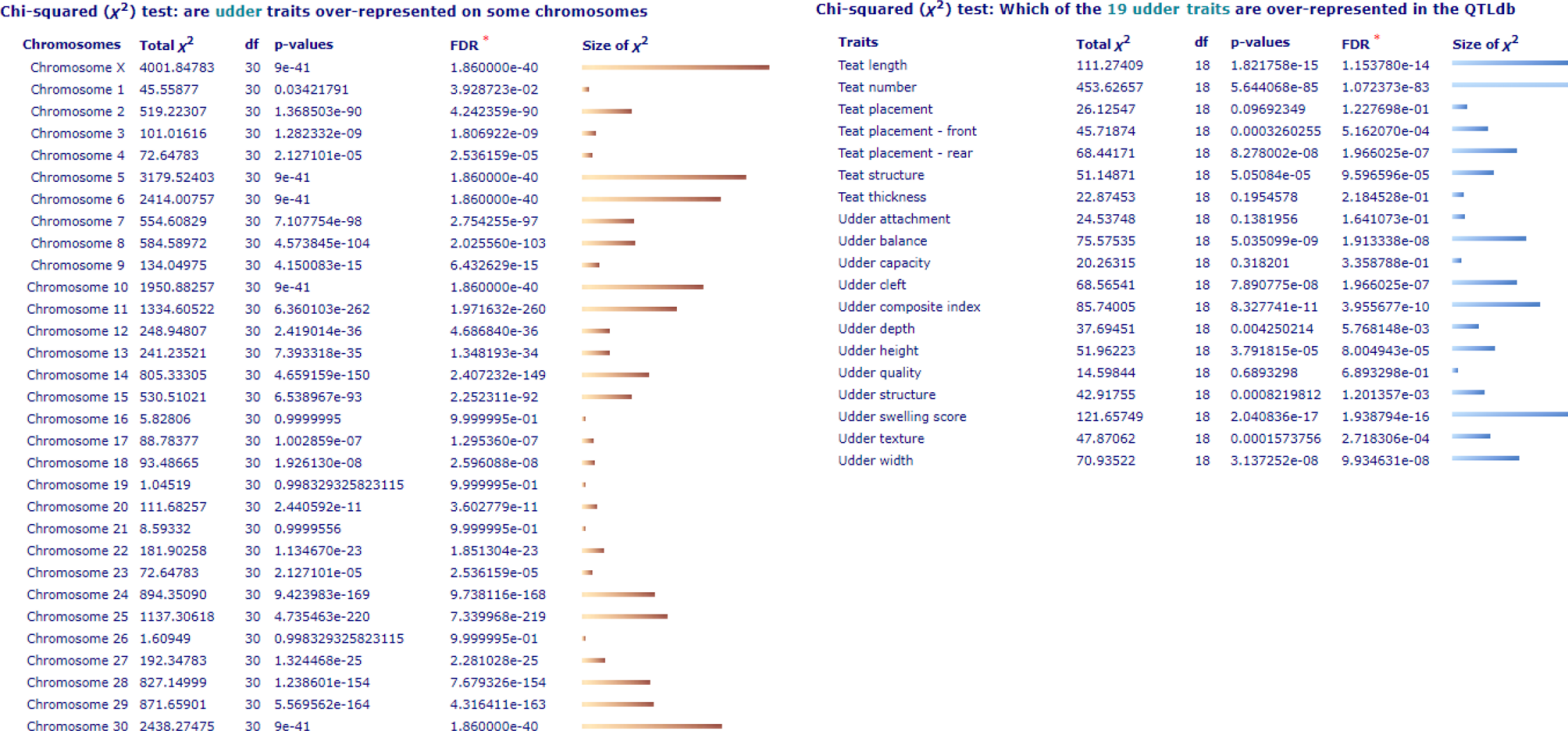
Whole genome analysis for QTL/association enrichment in cattle, including 4,040 QTLs associated with 19 udder traits spread on 31 chromosomes, utilizing QTL Data-Base. *FDR: is short for "false discovery rate", representing the expected proportion of type I errors. A type I error is where you incorrectly reject the null hypothesis, i.e. you get a false positive. It's statistical definition is FDR = E (V/R|R > 0) P(R > 0), where V = Number of Type I errors (false positives); R = Number of rejected hypotheses. Benjamini–Hochberg procedure is a practical way to estimate FDR.

**Figure 3:**
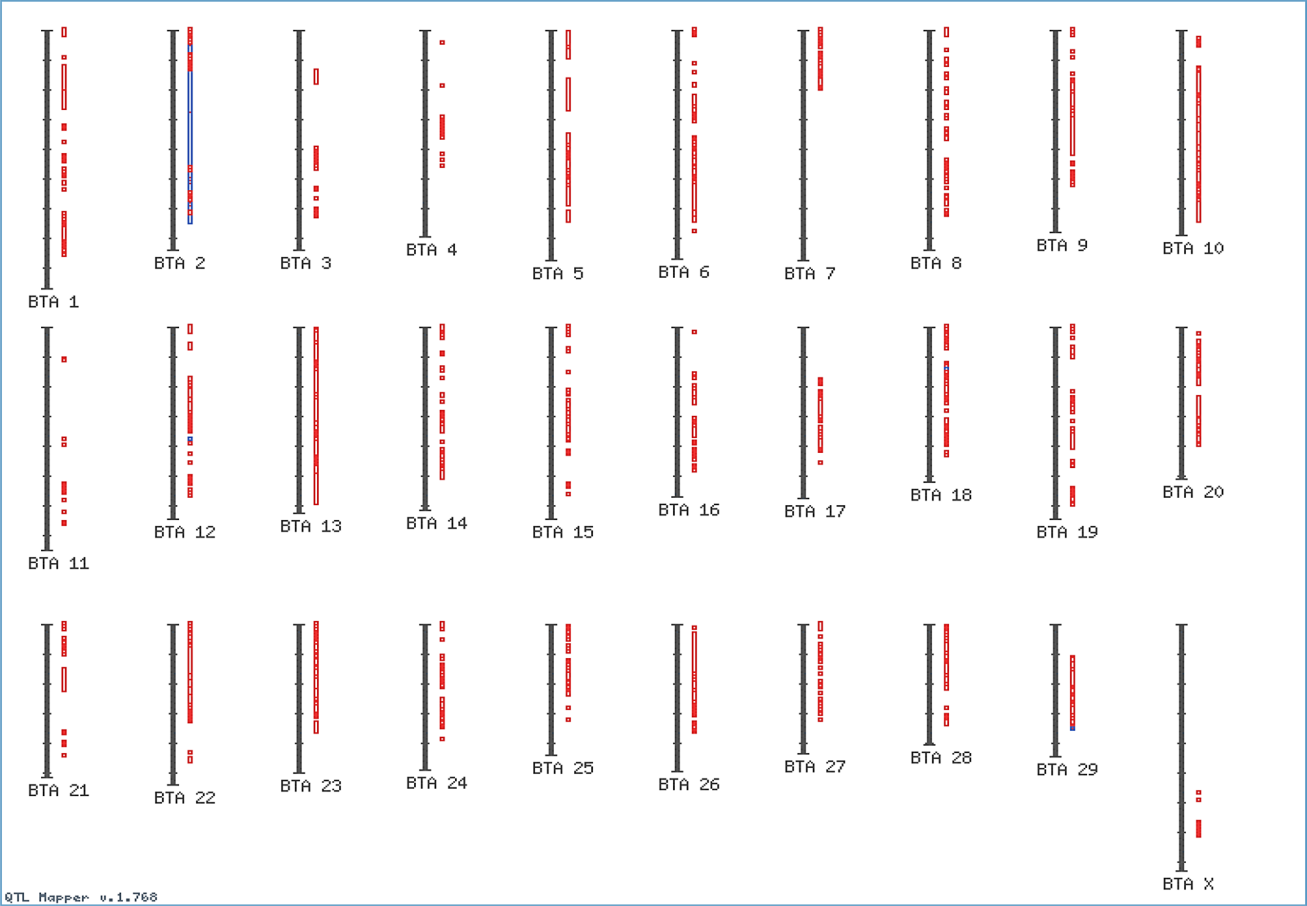
QTL/associations for udder characteristics in the Cattle Genome (It shows genome locations where an udder trait is mapped by QTL or SNP associations).

By investigating the QTL database for the cattle, the results revealed that there are 193,641 QTLs uncovered by 1122 different studies which were associated with 686 different economic traits. 4040 QTLs out of 193,641 were associated with udder traits (Table 8). These QTLs represent a huge raw material for future further studies as complementary to the current study on the same breed and individuals. Worth mentioning, Fig. 2 shows the whole genome analysis for 4,040 QTLs which were associated with 19 udder traits spread on 31 chromosomes in the cattle, while Fig. 3 shows locations where the udder traits are mapped by QTLs or candidate SNPs associations on different chromosomes in the cattle.

**Table 8:** Number of QTLs for many species based on animal QTL Database updated to 2023.

No	Species	Number of QTLs	Number of publications	Concerning traits
1	Cattle	193,641 QTLs	1122 publications	Represent: 686 different traits
2	Pig	35,846 QTLs	773 publications	Represent: 693 different traits
3	Chicken	18,313 QTLs	381 publications	Represent: 370 different traits
4	Sheep	4504 QTLs	236 publications	Represent: 267 different traits
5	Horse	2649 QTLs	107 publications	Represent: 65 different traits
6	Goat	129 QTLs	7 publications	Represent: 26 different traits

Top cattle QTL associations in the data-base 2023				
No.	Traits	Number of QTL		
1	Udder traits	4040		
2	Milk composition—fat	45,591		
3	Milk composition—protein	25,898		
4	General reproduction parameters	21,438		
5	Fertility	20,115		
6	Growth	15,237		
7	Sensory characteristics	11,577		
8	Milk production & yield	9108		
9	Anatomy	5013		
10	Semen quality	3803		
11	Fatness	3751		
12	Disease	3658		
13	Feed intake	3118		
14	Limb traits	2915		
15	Conformation	2899

## Discussion

The calculated coefficients of variation revealed low discrepancies among cows for udder characteristics which confirm a possible genetic control for udder conformation. Every udder shape has its own specific characteristics which impose an expressive effect on udder and teat parameters. The present results on udder circumference were similar to those reported by Mona and Semaida^43^ Sid Ahmed and El Barbary^44^ on cup shaped udder of Friesian cows, but the fore udder depths was lower than those of cup (29.2 cm), round (27.4 cm) and goaty (25.2 cm) udder shapes. Also, it was higher than 17.2 cm found in Romanian Black Spotted cows^45^. The hind udder depth results were much lower than 33.7 cm and 31.2 cm that reported for cup and round udder shapes of Friesian dairy cows, respectively^44^. The present result of udder rear depths for bowl udder shape (28.3 cm) was similar to that reported by Avarvarei^45^ in Romanian Black Spotted cows. The present results of teat measurements were similar to those of Deng et al.^46^. Milne^47^ revealed that morphology of the teat is recognized as part of the passive defence mechanism against intra-mammary infection, so short teats are more favourable for high milk producing cows than long teats.

The effects of parity and udder shape on udder and teat measurements in the current study were similar to those reported by Tilki et al.^28^ who reported that the udder measurements were affected by lactations number because udder tissue might be continuous to develop up to parity six then after starts to regress thereafter. This was confirmed by Singh et al.^48^ who reported that all udder and teat measurements had showed increasing trends up to the 5th parity. Consistently, Bhuiyan et al.^14^ reported that udder length increased gradually up to lactation six. Modh et al.^49^ reported an increasing trend at the rate of 24.3 and 9.7% in udder length and width, respectively of Gir cows at the first two parity, but afterwards the udder length became static while udder width became static up to parity four then exhibited 15.16% increased between parity four and five, whereas udder depth was at par. However, the mean udder depth increased from 19.4 to 28.5 cm, respectively in Vrindavani cattle^48^ similar to the present results. Modh et al.^49^ reported gradual increase in length, width and depth of the udder as the number of parity increases; multiparous cows had larger volume of udder than primiparous cows. The differences observed in the present study in udder length, width and depth in different parities were statistically significant (*P* < 0.05). Similar findings were reported by Patel^50^.

The current FTL and RTL were smaller than those reported by Modh et al.^49^ who obtained FTL and RTL of Gir cows in different parities ranging from 8.74 to 9.82 and 7.97 to 8.88 cm, respectively with gradual non-significant increase in length of fore (7.56%) and rear (8.28%) teat between parities one and two. Moreover, Patel et al.^51^ found significant differences observed between FTL and RTL in different parities of crossbred cows ranging from 5.48 to 6.52 cm and 4.92 to 5.94 cm, respectively with gradual increase in length by advancement of parity. Similar results were reported on Holstein, Vrindavani cattle, Hariana cows, Tharparkar cows, Kankrej cows and Gir cows by many workers^48,52^. With respect to teat diameters, Modh et al.^49^ found that the differences observed between fore and rear teat diameters in different parities were at par and no definite trend was detected with advancement of parity. Similar results were recorded by Sharma et al.^16^ on Hariana and Tharparkar cows. For teats length, Antalík and Strapák^53^ observed a gradual increase with advancement parity order. Similar to the current results, Tilki et al.^28^ and Antalík and Strapák^53^ reported that stage of lactation did not exert any effect on the udder or teat measurements.

The present parity effect on milk production traits was not significant. These results were in agreement with Atakan^54^ who found that parity had no significant effect on lactation length and 305-day milk yield of Friesian cows. On the contrary, Mellado et al.^55^ found that parity had significant influence on milk yield. Also, Lee and Kim^56^ reported that the differences in total milk yield among parities were significant. The contradicting results may be attributed to difference in analytical models, in herd size and the age of animals. However, the relationship between udder shapes and milk yield of cattle are well established. The present results are in agreement with those of Ghosh and Prasad^57^ and Bhuiyan et al.^14^ who obtained high milk yield from cows having bowl shaped udder compared to other udder shapes in cows. Also, Prasad et al.^58^ found that Murrah buffaloes bowl shaped udders produced the highest average daily milk yield followed by pendulous, globular and then goaty udders. Thus raising cows with bowel shaped udder may improve the efficiency of milk production.

The high positive genetic correlation of UW, UFD and URD with milk production activities were in agreement with these evaluated by various authors. Such high genetic correlations indicate that genetic selection for high milk yield will be associated with wider udders. In the current study and in previous literature for Holsteins ^59^ udder depth was the most unfavourably correlated with milk yield but the rear udder height and width were the most favourably related to milk yield. Also, Berry et al.^60^ reported mild positive genetic correlation (0.36) between udder support and milk yield. DeGroot et al.^61^ reported negative genetic correlations (− 0.45) between fore-udder attachment and milk yield. On the contrary, Samoré et al.^62^ found favourable correlation between strong fore-udder attachments and high yield of Brown Swiss data.

High genetic correlations between the teat diameter and milk production indicate that genetic selection for milk yield will establish cows with substantial teat size. However, weak correlations were estimated between front teat placement, teat length, rear teat position and central ligament with milk yield ^63^. Mavrogenis et al.^64^, reported that the relationships between teat measures and milk production were generally low. Also, Otwinowska-Mindur et al.^65^ found that the genetic correlations of persistency measures with rear teat placement were rather low negative. this should be regarded as favourable, because selection for better persistency would decrease the scores for rear teat placement towards the optimum of this trait.

In this study, heritability estimates for udder and teat measurements were low to moderate. Low estimates of h^2^ for udder and teat measurements with milk production traits concluded that environmental variation contributed the major part of the total variation for the milk production traits, thus management may be an effective factor in improving such traits. In addition, the heritability estimates for udder and teat measurements can change without any genetic change occurring when the environment starts contributing to more variation. In the previous studies, heritability recorded for rear udder height was − 0.77^59^ and 0.31^61^. Špehar et al.^66^ obtained a heritability value of 0.14 for fore udder attachment. The heritability of the teat diameter in this study was similar to that reported by Seykora and McDanel^17^ which was 0.44 and that of teat length was equal to Tapki and Guzey^59^ estimate.

In this study, UH and RTL having low heritability declared the importance of environmental and non-additive genetic effects on alteration of these traits. However, the traits moderate to high heritability estimates may show response for direct selection and reasonable correlation response for other traits. The magnitudes of these values indicate that a considerable proportion of the phenotypic variation occurs due to differences in genes with additive effects and that genetic gain in response to selection procedures might occur. Heritability values of udder characteristics in various studies show great variability depending on breed and housing and scoring system^67^.

The current UW having the highest correlation coefficients and R^2^ was the most suitable indicator for predicting milk traits follow by udder front and rear depth. None of the teat measurements could be used efficiently for predicting milk yield in this dataset. Deng et al.^46^ obtained relatively high positive correlation coefficient (0.64) between milk yield and udder length and concluded that the latter should be an important criterion for selecting dairy cows and can be used with a satisfactory precision for predicting milk yield because the regression of milk yield on udder length had the highest R^2^and each cm increase in udder length represent 0.22 kg extra yield of milk. Sinha et al.^68^ reported 0.51, 0.51 and 0.55 coefficients of determinations for the regressions of total milk yield, 305 days milk yield and peak yield, respectively on udder width and Singh et al.^48^ reported higher accuracy of prediction for the regression of milk yield on udder width measurement.

Concerning the genetic background of udder characteristics, the heritability of udder and teat measurements/characteristics is moderate (0.23–0.45), which in turn facilitates the genetic improvement programmes^69^. Genomic regions related to the udder and teat traits were reported in several dairy cattle^38^. Numerous reliable studies uncovered many QTLs and their association with udder and teat structure, as economic and significant phenotypes (Table 8, Figs. 2 and 3). In this aspect, 15 SNP loci on *BTA-5* were related to udder support scores in the cattle. One out of these 15 was associated with average teat diameter^70^. Additionally, Flury^71^ confirmed that seven udder conformation traits were correlated with five genomic regions on *BTA-3*, *-5*, *-6*, *-17*, and *-25* in Brown Swiss cattle. Where, several significant SNPs on *BTA-6* were associated with teats diameter and fore udder length. While height-significant SNPs in the coding-region of *SNX-29* gene were related to trait rear udder. Moreover, many significant SNPs on *BTA-17* (62 Mb) were related to front teat placement, rear teat placement and rear udder width. Also, Marete et al.^72^ reported that 10 candidate genes were uncovered for their associations with udder traits, including *RREB1*, *FGF2*, *FGFR-2*, *ESR-1*, *IQGAP-3*, *GLI-2*, *PGR*, *BTRC*, *TGFBR-2* and *PRLR*, in French dairy cattle. Furthermore, 18 candidate SNPs within *STXBP-6* and *SLF-1* genes were related to five udder traits in the Chinese Holstein breed^38^. Meanwhile, several SNPs within *LGALS-2*, *GCLC* and *ADGRB-3* genes were related to udder depth trait in Holstein cattle^38,73^.

On the other side, udder traits are related to sustainable milk production^74^, mastitis resistance^75^ and longevity^76^. Thus, there is a great benefit to considering and investigating these traits side by side with their genetic background in future studies.

## Conclusion

Result of the present study shows that parity had significant effect on udder and teat characteristics. Udder shape has a considerable relationship and affects milk yield and lactation length. The bowel udders shape produces high milk yield and associate with long lactation length compared to other udder shapes, therefore, should be considered while selecting Friesian cows. The genetic correlation of udder, teat measurements with milk yield indicated a correlated response; therefore, selection to improve teat diameter and udder width should increase milk yield. All udder and teat characteristics could be included in a selection index criterion (except UH and RTL) to improve the total merit of lactating cows directly or through correlated response with other traits. Also, udder width and udder rear depth can be used as a good indicator for predicting milk yield and lactation length. Finally, the detected QTLs, candidate genes and significant SNPs are potential tools to improve milk productivity side by side with the udder phenotypic investigations.

## Data Availability

All data generated or analyzed during this study are included in this manuscript.
